# NPS in MCI: A scientometric analysis

**DOI:** 10.1002/alz70857_096008

**Published:** 2025-12-24

**Authors:** Arisara Amrapala

**Affiliations:** ^1^ Faculty of Medicine, Chulalongkorn University, Pathumwan, Bangkok, Thailand; Center of Excellence in Digital and AI for Mental Health (AIMET), Faculty of Engineering, Chulalongkorn University, Pathumwan, Bangkok, Thailand

## Abstract

**Background:**

A scientometric study of the existing research on behavioral and psychological symptoms of dementia (BPSD) in mild cognitive impairment (MCI) was conducted to obtain key trends, influential networks, and major contributors in the field.

**Method:**

Relevant literature was retrieved from the Web of Science database, with full‐text and citation records extracted for analysis. Author and journal information was retrieved using the Bibliometrix R package; CiteSpace was used to compute the co‐citation analysis, co‐occurrence analysis, and collaboration networks; and VOS‐viewer was employed to visualize network maps of the most‐cited journals and co‐occurring author keywords network.

**Result:**

Our search found 12,369 studies published between 1980 and 2022 that satisfied the inclusion criteria. Co‐citation analysis unveiled 51 highly credible clusters (significant modularity score of 0.856 and silhouette score of 0.932), with the largest cluster encompassing ‘cognitive disorders’. Five general research domains were derived from the network: symptoms, diagnosis, brain substrates, biochemical pathways, and interventions. Data from recent years highlighted ‘depression’ as the most cited keyword, while current research focused on gut microbiota, e‐health, COVID‐19, cognition, and delirium. The USA and UK had the highest influence and citations, and the PRC exhibited notable growth.

**Conclusion:**

This scientometric approach provides a comprehensive overview of the research evolution, emerging trends, and key focus areas in BPSD in MCI. These findings offer valuable insights for researchers and clinicians, aiding in evidence‐based decision‐making and enhancing the quality of future research in this domain.

